# Negative impact of chemotherapy on kinetic growth rate of the future liver remnant if applied following PVE or ALPPS

**DOI:** 10.1371/journal.pone.0307937

**Published:** 2025-03-07

**Authors:** Klara Welcker, Martin A. Schneider, Tim Reese, Andrea Ehrenfeld, Hauke Weilert, Axel Stang, Peter Wohlmuth, Mia-Maria Warnke, Carolin Reiner, Thomas von Hahn, Karl J. Oldhafer, Andreas H. Mahnken, Roland Brüning

**Affiliations:** 1 Radiology and Neuroradiology, Asklepios Hospital Barmbek, Hamburg, Germany; 2 Department of Surgery, Division of Liver-, Bileduct- and Pancreatic Surgery, Asklepios Hospital Barmbek, Hamburg, Germany; 3 Gastroenterology, Hepatology and interventional Endoscopy, Asklepios Hospital Barmbek, Hamburg, Germany; 4 Oncology, Asklepios Hospital Barmbek, Hamburg, Germany; 5 Medical Faculty, Semmelweis University Budapest, Hamburg, Germany,; 6 Biostatistics, ProResearch, Hamburg, Germany; 7 Clinic for Diagnostic and Interventional Radiology, Philipps University and University Clinic Marburg, Marburg, Germany; Cincinnati Children’s Hospital Medical Center, UNITED STATES OF AMERICA

## Abstract

**Purpose:**

Modern liver surgery has improved the percentage of potentially resectable malignant tumors. However, if the future liver remnant is small, patients remain at risk of developing postoperative liver failure. Thus, the future liver remnant must be increased, while at the same time, the primary tumor may have to be controlled by chemotherapy. To address this conflict, we retrospectively analyzed the changes in hypertrophy before and after Associating Liver Partition with Portal vein ligation for Staged hepatectomy (ALPPS) or Portal Vein Embolization (PVE), with or without parallel systemic chemotherapy.

**Materials and Methods:**

We retrospectively analysed 172 patients (54 female and 118 male), treated with ALPPS in 90 patients (median age 61 years [Q1, Q3: 52,71]) and with PVE in 82 patients (median age 66 years [Q1, Q3: 56,73]). The median control interval was 4.9 [Q1, Q3: 4.0, 6.0] weeks after the PVE, and 2.6 [Q1, Q3: 1.6, 5.8] weeks after ALPPS step 1.

**Results:**

The overall kinetic growth rate (median) for the entire group was 0.02 (2%) per week. When systemic chemotherapy was administered prior to intervention, the kinetic growth rate of these treated patients (vs. untreated) exhibited a median of 0.020 [Q1, Q3: 0.011, 0.067] compared to 0.024 [Q1, Q3: 0.013, 0.041] (p = 0.949). When chemotherapy was administered after the PVE/ ALPPS treatment, the kinetic growth rate declined from a median of 0.025 [Q1, Q3: 0.013, 0.053] to 0.011 [Q1, Q3: 0.007, 0.021] (p = 0.005). Subgroup analysis showed statistically significant effects only in the PVE group (median ALPPS -45% (p = 0.157), PVE -47% (p = 0.005)).

**Conclusion:**

This retrospective analysis indicated that systemic chemotherapy given after PVE/ the first step of the ALPPS procedure, i.e., the growth phase, has a negative effect on the kinetic growth rate.

## Introduction

Modern techniques in hepatobiliary surgery of the liver have improved the proportion of potentially resectable malignant liver tumors [1]. However, if the proportion of the liver volume remaining in situ after surgery [2] is small, patients remain at risk of developing postoperative liver failure [1,3]. To address this issue, most centers routinely measure the future liver remnant (FLR) before planning surgery. Depending on the underlying disease, an FLR volume of less than 30 to 40% has been postulated as a predictor of postoperative liver dysfunction [4,5].

If required, an FLR below this percentage can be increased by hypertrophy strategies, either surgically or through interventional radiological procedures. The two most established methods are Associating Liver Partition with Portal vein ligation for Staged hepatectomy (ALPPS) [6,7] and Portal Venous Embolization (PVE) [2,8], both of which are techniques to redirect portal blood flow to the non-embolized/ untreated segments remaining after resection [9]. Both methods have specific strengths and weaknesses, and the growth rate of hypertrophy and complications are different and need to be considered. There is a consensus that the required future liver volume is primarily dependent on the quality of the liver tissue [2,10,11].

However, during this interval towards hypertrophy, it may be necessary to control the primary tumor to prevent further expansion. Systemic chemotherapy is often used to control tumor size, but this may come at the expense of the rate of hypertrophy. Unfortunately, there is only limited, and conflicting data on the impact of chemotherapy on growth rate and hypertrophy.

To address this issue, we retrospectively analysed data from our center using both methods, ALPPS and PVE. We dichotomised patients into groups based on whether they received chemotherapy before or during the hypertrophy phase, and those who did not receive chemotherapy. We used the kinetic growth rate (KGR) as the most established marker to compare effects.

## Materials and methods

### Patient selection and chemotherapy

A total of 185 interventions (90 ALPPS and 95 PVE) performed between 2013 and 2020 were identified retrospectively from our hospital database. The inclusion criteria were primary or secondary neoplastic liver lesions, liver- dominant disease, and a positive tumor board vote towards a hypertrophy model and planned (right) extended hemihepatectomy. The analysis included 90 ALPPS patients and 82 patients who received PVE as the sole intervention before resection. To ensure clarity regarding growth rates, a further 12 patients who also received hepatic vein embolization (HVE) in addition to PVE were excluded. The analysis also excluded one patient who received transarterial chemoembolization (TACE) in addition to PVE during the same session, at the discretion of the interventionalist. Chemotherapy regimens administered more than six months prior to the PVE/ first part of ALPPS intervention were excluded from the analysis, as well as chemotherapy administered after the operation following PVE/ second part of ALPPS. Patient details can be found in Tables 1–2 and Table 3.

**Table 1:** Patient data.

Characteristic	Overall, N = 172^1^	PVE, N = 82	ALPPS, N = 90
Age (years)			
Median [Q1, Q3]	63 [54, 72]	66 [56, 73]	61 [52, 71]
Mean (SD)	62 (12)	64 (11)	60 (13)
Effect size	0.164		
Gender			
Female	54 (31)	29 (35)	25 (28)
Male	118 (69)	53 (65)	65 (72)
Effect size	0.082		
BMI			
Median [Q1, Q3]	24.6 [22.5, 28.4]	24.5 [22.2, 27.8]	24.7 [22.8, 28.6]
Mean (SD)	25.8 (4.8)	25.8 (5.3)	25.9 (4.3)
Effect size	0.011		
Chemotherapy			
Treated with, overall	83 (48)	32 (39)	51 (57)
Pre-intervention	79 (46)	30 (37)	49 (54)
Post-intervention	22 (13)	11 (13)	11 (12)
Treated without, overall	89 (52)	50 (61)	39 (43)
Pre-intervention	93 (54)	52 (63)	41 (46)
Post-intervention	150 (87)	71 (87)	79 (88)
Chemotherapy (pre-intervention)			
FOLFIRI	24 (14)	4 (4.9)	20 (22)
FOLFOX	18 (10)	10 (12)	8 (8.9)
FOLFOXIRI	24 (14)	10 (12)	14 (16)
Others	13 (7.6)	6 (7.3)	7 (7.8)
None	93 (54)	52 (63)	41 (46)
Effect size	0.271		
Chemotherapy (post-intervention)			
FOLFIRI	2 (1.2)	0 (0)	2 (2.2)
FOLFOX	11 (6.4)	3 (3.7)	8 (8.9)
FOLFOXIRI	5 (2.9)	4 (4.9)	1 (1.1)
Others	4 (2.3)	4 (4.9)	0 (0)
None	150 (87)	71 (87)	79 (88)
Effect size	0.243		
Chemo cycles (pre-intervention)			
Median [Q1, Q3]	0.0 [0.0, 5.0]	0.0 [0.0, 3.0]	2.0 [0.0, 5.8]
Mean (SD)	2.8 (3.8)	2.1 (3.6)	3.3 (3.9)
Effect size	0.189		
Chemo cycles (post-intervention)			
0	150 (87)	71 (87)	79 (88)
1	2 (1.2)	1 (1.2)	1 (1.1)
2	10 (5.8)	2 (2.4)	8 (8.9)
3	5 (2.9)	5 (6.1)	0 (0)
4	1 (0.6)	1 (1.2)	0 (0)
≥ 5	2 (1.2)	1 (1.2)	1 (1.1)
≥ 20	2 (1.2)	1 (1.2)	1 (1.1)
Effect size	0.018		
Chemotherapy cycles (post-intervention) scaled to KGR interval			
Median [Q1, Q3]	0.00 [0.00, 0.00]	0.00 [0.00, 0.00]	0.00 [0.00, 0.00]
Mean (SD)	0.19 (0.58)	0.16 (0.45)	0.22 (0.68)
Effect size	0.005		
Kinetic growth rate			
Median [Q1, Q3]	0.02 [0.01, 0.05]	0.02 [0.01, 0.03]	0.04 [0.02, 0.09]
Mean (SD)	0.04 (0.05)	0.02 (0.02)	0.06 (0.06)
Effect size	0.414		
Future liver remnant pre-intervention (ml)			
Median [Q1, Q3]	379 [268, 489]	418 [287, 520]	339 [267, 477]
Mean (SD)	397 (169)	419 (168)	378 (167)
Effect size	0.122		

^1^c (“Median [Q1, Q3]”, “Mean (SD)”); n (%)

**Table 2:** Histology of tumor and parenchymal liver tissue.

Characteristic	Overall, N = 172^1^	PVE, N = 82	ALPPS, N = 90
Tumor histology			
Colorectal liver metastases (CRLM)			
Overall	94 (55)	34 (41)	60 (67)
FOLFOX	23 (13)	13 (16)	10 (11)
FOLFIRI	23 (13)	4 (5)	19 (21)
FOLFOXIRI	23 (13)	8 (10)	15 (17)
Others	6 (3)	2 (2)	4 (4)
No chemotherapy	19 (11)	7 (9)	12 (13)
Cholangiocellular carcinoma (CCA)			
Overall	39 (23)	30 (37)	9 (10)
FOLFOX/ FOLFIRI/ FOLFOXIRI	0 (0)	0 (0)	0 (0)
Others	3 (2)	3 (4)	0 (0)
No chemotherapy	36 (21)	27 (33)	9 (10)
Other types of tumor^2^			
Overall	40 (23)	19 (23)	21 (23)
Others	5 (3)	2 (2)	3 (3)
No chemotherapy	35 (20)	17 (21)	18 (20)
Histology of parenchymal liver tissue			
Liver fibrosis			
Yes	23 (13)	13 (16)	10 (11)
No/ not specified	74 (43)/ 75 (44)	36 (44)/ 33 (40)	38 (42)/ 42 (47)
Hepatic steatosis			
Yes	15 (9)	8 (10)	7 (8)
No/ not specified	80 (47)/ 77 (45)	41 (50)/ 33 (40)	39 (43)/ 44 (49)
Liver cirrhosis			
Yes	2 (1)	1 (1)	1 (1)
No/ not specified	155 (90)/ 15 (9)	71 (87)/ 10 (12)	84 (93)/ 5 (6)

^1^c (“Median [Q1, Q3]”, “Mean (SD)”); n (%)

^2^other types of tumor: gallbladder carcinoma, hepatocellular carcinoma (HCC), pancreatic cancer, giant hepatic haemangioma, breast cancer, sarcoma (GIST, Ewing, ESL, LMS), calcifying fibrous tumor (CFT), renal cell carcinoma (RCC), adrenal tumor, malignant melanoma and unknown.

**Table 3:** Anatomical features of the tumor-affected liver parenchyma.

Characteristic	Overall, N = 172^1^	PVE, N = 82	ALPPS, N = 90
Distribution pattern			
Unifocal	33 (19)	17 (21)	16 (18)
Multifocal	123 (71.5)	51 (62)	72 (80)
None of them	16 (9)	14 (17)	2 (2)
Affected liver lobe and segment			
Left lobe	110 (64)	46 (56)	64 (71)
Segment I	8 (5)	1 (1)	7 (8)
Segment II	33 (19)	9 (11)	24 (27)
Segment III	29 (17)	9 (11)	20 (22)
Segment IVa/ b	96 (56)	42 (51)	54 (60)
Right lobe	151 (88)	65 (79)	86 (96)
Segment V	113 (66)	50 (61)	63 (70)
Segment VI	99 (58)	40 (49)	59 (66)
Segment VII	108 (64)	39 (48)	69 (77)
Segment VIII	123 (72)	52 (63)	71 (79)
None of them	16 (9)	14 (17)	2 (2)
Extent of largest tumor lesion (mm)			
Median [Q1, Q3]	45 [25, 84]	53 [149, 86]	41 [25, 78]
Mean (SD)	56 (39)	57 (36)	55 (41)
Effect size	0.026		

^1^c (“Median [Q1, Q3]”, “Mean (SD)”); n (%)

The criteria for choosing ALPPS or PVE were not identical and there was a lack of randomization. In addition to pre- and post- interventional CT scans, laboratory parameters and biometric values, the following data were collected: age, gender, intervention timeline (including the date of the PVE and operation/ dates of the first and second ALPPS), information about the chemotherapy administered (timeframe, number of cycles, type of chemotherapy) and the calculated KGR.

All data on chemotherapy were obtained from the hospital’s digital patient record M-KIS® (Meyerhofer, Germany), including conference protocols and discharge letters. The patients were anonymised after primary viewing of the patient data. The retrospective study design was approved by the Ethics Committee of the Hamburg Medical Association (processing number 2023-300389-WF). Patients who received chemotherapy were divided into two sub-groups: “chemotherapy before PVE/ ALPPS” and “chemotherapy after PVE/ ALPPS”. The former included patients who received chemotherapy before the PVE/ first part of ALPPS intervention, while the latter included patients who received chemotherapy between the PVE/ first part of ALPPS intervention and the hemihepatectomy after PVE/ second part of ALPPS. The patients who received chemotherapy before and after the hypertrophy procedure were divided into subgroups by splitting up the chemotherapy cycles.

Patients who received chemotherapy were further divided into additional groups based on their primary chemotherapy regimen in the first cycle including FOLFOX, FOLFIRI, FOLFOXIRI, and “others”. If data were missing regarding the chemotherapy given, cycles or time periods, missing data were added using a statistical similarity relationship. The category “others” includes the types of chemotherapy that cannot be classified as FOLFOX, FOLFIRI or FOLFOXIRI, such as alkylating agents (Mitomicin, Cyclophosphamid, Cisplatin, Carpoplatin, Oxaliplatin), pyrimidine analogues (Gemcitabin, 5-Fluoruracil, Capecitabin), anthracyclines (Epirubicin), taxane derivates (Paclitaxel), VEGF inhibitors (Bevacizumab), tyrosine kinase inhibitors (Imatinib), SERMs (Tamoxifen) and gonaderolin analogues (Goserelin).

### Volumetry

The KGR was calculated using volumetric data from pre- and post-intervention CT scans. Please refer to Table 1 for more details.

### Intervention

#### PVE.

PVE was performed at our centre under general anaesthesia and was carried out by an interventional radiologist with more than 15 years of experience plus an experienced sonographer. Using ultrasound-guided transcutaneous transhepatic access (Toshiba Aplio XV, Toshiba Europe GmbH, Neuss, Germany), we secured the peripheral portal branch with a 6 French sheath.

To protect the FLR, we used ipsilateral (right-sided) access. A Portography was performed in three planes (typically 0, −  25, and +  25 degrees rotation). We then accessed the right portal vein branches for embolization using a five or six French “reverse” S1 Sidewinder catheter (Cook Medical, Bloomington, IN); this technique has been previously described in detail [12]. The embolization material was chosen by the responsible interventionist; either Polyvinyl alcohol particles (Contour™ PVA particles, 250–355 mm; Terumo, Tokyo, Japan) in combination with fibred coil (mREYE® spiral sizes 3–12 mm, Cook Medical, see above), or a mixture of Lipiodol® (Guerbet, France) and synthetic surgical glue (Gluebran®, GEM Viareggio Italy). The procedure was terminated if total occlusion of target vessels was observed by portography.

#### ALPPS.

The first step in the ALPPS procedure commenced with a bilateral subcostal laparotomy [13] to fully mobilise the right liver from the caval vein. Following ligation of the right portal vein, a parenchymal dissection of the liver parenchyma was performed to varying degrees along the falciform ligament [14].

If the postoperative CT scan indicated a sufficient volume of FLR, the second step of the ALPPS procedure was carried out. This re-laparotomy involved dividing the bile duct, right and middle hepatic veins, and resecting the tumor-bearing extended right lobe. Any remaining parenchymal bridges of liver tissue, if presented were separated [14].

### Control-interval

The interval of hypertrophy in PVE patients was defined as the interval between the PVE and the control CT. For ALPPS patients, the interval of hypertrophy was determined by the time between the pre-intervention CT, before the first step of ALPPS, and the control CT immediately before the second intervention part of ALPPS. After PVE, the interval was 4.9 weeks (median [Q1, Q3: 4.0, 6.0]), and after the first step of ALPPS, was 2.6 weeks (median [Q1, Q3: 1.6, 5.8]).

### Volumetric evaluation

Venous-phase contrast-enhanced multi-slice CT scanners (GE Optima 660 (GE Healthcare, Pittsburgh, PA) or Philips Brilliance 128 (Philips Healthcare, Best, Netherlands)), with a slice thickness of 5 mm, were used for imaging and volumetry. Volumetric post-processing was performed using the advantage workstation 4.1.2 (GE Healthcare) by manually outlining Segments II and III, with the aid of threshold values. We excluded post surgical remnants and non-parenchymal structures such as cysts, portal branches, or bile ducts by thresholding the outlined volume, if necessary. Prior to each surgical step, all patients underwent a volumetric evaluation of the FLR, which was calculated by outlining the remaining segments of the liver on axial planes.

The total estimated liver volume (TELV) was calculated using the formula of Vauthey et al. [15], based on the body surface area (BSA) calculated by Mosteller [16]. The degree of hypertrophy (DH) was calculated as described by Shindoh et al. [17]. Future liver remnant volume (FLRV) and standardized future liver remnant (sFLR) were calculated and captured for each patient before surgery, following the method as described by Vauthey et al. [15]. The calculation of KGR for patients with PVE involved dividing DH at the first post-PVE volume assessment (expressed as a percentage) by the time elapsed since PVE (measured in weeks) for the same assessment [17]. In patients who undergo ALPPS as their interventional treatment, KGR is defined as the degree of hypertrophy divided by the time between the first step of ALPPS and CT prior to the second step of ALPPS, measured in weeks [15,17].

### Statistical methods

Continuous data were summarized as means ±  standard deviations or as medians [25th and 75th percentiles] as appropriate. Categorical data were presented as N (%). Differences in KGR between patients who received chemotherapy and those who did not were presented as boxplots and analysed using the Wilcoxon Mann-Whitney test. The effect size (r) was calculated as the Z statistic of the Wilcoxon Mann-Whitney test divided by the square root of the sample size (N). The r- value ranged from 0 to 1. An r value of 0.10 to less than 0.3 was considered a small effect, 0.30 to less than 0.5 was considered a moderate effect, and 0.5 or greater was considered a large effect. All p-values were two-sided, and a p- value of less than 0.05 was considered significant. All calculations and figures were performed using the statistical analysis software R [18]. The effect sizes were reported with point estimates and 95% confidence intervals and the classification by Funder et al. [19] was used. Interpretations of effect sizes were determined as follows: r <  0.05 considered tiny, 0.05 ≤  r <  0.1 very small, 0.1 ≤  r <  0.2 small, 0.2 ≤  r <  0.3 medium, 0.3 ≤  r <  0.4 – large, and r ≥  0.4- was very large [19].

## Results

### Cohort characteristics

The retrospective study analysed 172 patients, with 90 receiving ALPPS and 82 receiving PVE as a therapeutic procedure. The median age of the entire group was 63 years (effect size 0.164, PVE group 66 years, ALPPS group 61 years), with 54 female patients overall. The PVE treated group included 29 women, while the ALPPS group included 25 women. The median body mass index for the entire group was 24.6; for the PVE group it was 24.5, and for the ALPPS group it was 24.7 (refer to Table 1).

Of the entire patient cohort, 83 patients received chemotherapy and 89 patients did not. Of the 83 patients who received systemic chemotherapy, 79 received it before and 22 after the intervention. Of the patients treated with ALPPS, a total of 51 patients received chemotherapy, while 39 patients did not receive any chemotherapy. Among these 51 patients, 49 patients received chemotherapy before the intervention and 11 received it after. In the PVE group, 32 patients received chemotherapy regardless of whether it was before or after intervention, while 50 patients did not receive any chemotherapy. In the PVE group, 30 patients received pre-intervention chemotherapy and 11 patients received post-intervention chemotherapy (Table 1).

The patients who received pre-intervention chemotherapy were divided into the following subgroups: FOLFIRI 24 (PVE group 4, ALPPS group 20), FOLFOX 18 (PVE group 10, ALPPS group 8), FOLFOXIRI 24 (PVE group 10, ALPPS group 14), others 13 (PVE group 6, ALPPS group 7), and none 93 (PVE group 52, ALPPS group 41) (effect size 0.271). The patients who received post-intervention chemotherapy were divided into the following subgroups: FOLFIRI 2 (PVE group 0, ALPPS group 2), FOLFOX 11 (PVE group 3, ALPPS group 8), FOLFOXIRI 5 (PVE group 4, ALPPS group 1), others 4 (PVE group 4, ALPPS group 0), none 150 (PVE group 71, ALPPS group 79) (effect size 0.243).

The number of chemotherapy cycles after intervention in relation to the number of patients was distributed as follows: zero cycles: 150 (PVE group 71, ALPPS group 79), one cycle: 2 (PVE group 1, ALPPS group:1), two cycles: 10 (PVE group 2, ALPPS group 8), three cycles: 5 (PVE group 5, ALPPS group 0), four cycles: 1 (PVE group 1, ALPPS group 0), five cycles: 2 (PVE group 1, ALPPS group 1), 20 cycles: 2 (PVE group 1, ALPPS group 1) (effect size 0.018.) See Table 1 for further details.

The median number of pre-intervention chemotherapy cycles was 0.0 overall, 0.0 in the PVE group, and 2.0 in the ALPPS group (effect size 0.189). The median of the post-intervention-chemotherapy cycles scaled to the KGR interval was 0.00 in total (PVE group 0.00, ALPPS group 0.00) and the mean was 0.19 in total (PVE group 0.16, ALPPS group 0.22) (effect size 0.005). The total KGR (median) in the whole group was 0.02 per week (PVE group 0.02, ALPPS group 0.04). The mean for the whole group was 0.04 (PVE group 0.02, ALPPS 0.06) (effect size 0.414). The median of pre-intervention FLR was 418 ml in the PVE group and 339 ml in the ALPPS group. The mean was 419 ml in PVE and 378 ml in the ALPPS group (effect size 0.122) (see Table 1).

### Tumor histology

In 94 patients of the total cohort (n = 172), the histology was colorectal liver metastases (CRLM). Of these 94 patients, 60 patients received ALPPS and 34 patients received PVE as their interventional treatment. The number of patients with CRLM assigned to each chemotherapy group was as follows: FOLFOX 23 (PVE group 13, ALPPS group 10), FOLFIRI 23 (PVE group 4, ALPPS group 19), FOLFOXIRI 23 (PVE group 8, ALPPS group 15), other 6 (PVE group 2, ALPPS group 4). 19 patients with CRLM did not receive any chemotherapy (PVE group 7, ALPPS group 12). Cholangiocellular carcinoma (CCA) was histologically confirmed in 39 patients in the whole group, 30 of whom were in the PVE group. The number of patients with CCA assigned to each chemotherapy group was as follows: FOLFOX, FOLFIRI and FOLFOXIRI 0, other 3 (PVE group 3, ALPPS group 0). 36 patients with CCA received no chemotherapy at all (PVE group 27, ALPPS group 9). Other tumor types were detected in 40 patients of the total collective (PVE group 19, ALPPS group 21). Gallbladder carcinoma, hepatocellular carcinoma (HCC), pancreatic cancer, giant hepatic haemangioma, breast cancer, sarcoma (gastrointestinal stromal tumor (GIST), ewing, embryonal undifferentiated sarcoma of the liver (ESL), leiomyosarcoma (LMS)), calcifying fibrous tumor (CFT), renal cell carcinoma (RCC), adrenal tumor, malignant melanoma and unknown tumor belong to the group mentioned above. In the rare case where two tumor types were identified histologically, both were noted and counted (see Table 2).

### Histology of parenchymal liver tissue

Histological examination of the removed liver tissue revealed fibrotic tissue in 23 patients (PVE group 13, ALPPS group 10), steatosis tissue in 15 patients (PVE group 8, ALPPS group 7) and cirrhotic tissue in 2 patients (PVE group 1, ALPPS group 1). Fibrotic tissue was not found in 74 patients (PVE group 36, ALPPS group 38), steatosis was not detected in 80 patients (PVE group 41, ALPPS group 39) and cirrhotic tissue was not observed in 155 patients (PVE group 71, ALPPS group 39). No further information was available on fibrosis in 75 patients (PVE group 33, ALPPS group 42), on steatosis in 77 patients (PVE group 33, ALPPS group 44) and on cirrhosis in 15 patients (PVE group 10, ALPPS group 5) (see Table 2).

### Anatomical features of the tumor-affected liver parenchyma

A unifocal distribution pattern of tumor tissue was observed in 17 of 82 patients in the PVE group and 16 of 90 patients in the ALPPS group. A multifocal distribution pattern was seen in 51 patients in the PVE group and 72 patients in the ALPPS group. 14 patients in the PVE group and two patients in the ALPPS group could not be classified into this distribution pattern (see Table 3).

Outside segments V to VIII, tumor tissue was detected in 110 patients of the entire collective (PVE group 46, ALPPS group 64), with segment IVa/b being mainly affected: 96 of 110 patients (PVE group 42, ALPPS group 54). In the right liver lobe, tumor tissue was detected in 151 patients of the total collective (PVE group 65, ALPPS group 86). The segments of the right liver lobe affected by tumor tissue were as follows: segment V 113 patients (PVE group 50, ALPPS group 63), segment VI 99 patients (PVE group 40, ALPPS group 59), segment VII 108 patients (PVE group 39, ALPPS group 69) and segment VIII 123 patients (PVE group 52, ALPPS group 71). 16 patients of the total collective could not be classified (see Table 3).

The median extent of the largest tumor lesion (in mm) was 45 (PVE group 53, ALPPS group 41). The mean was 56 (PVE group 57, ALPPS group 55) (effect size 0.026, see Table 3).

### Influence on hypertrophy in the entire group

**Fig 1:**
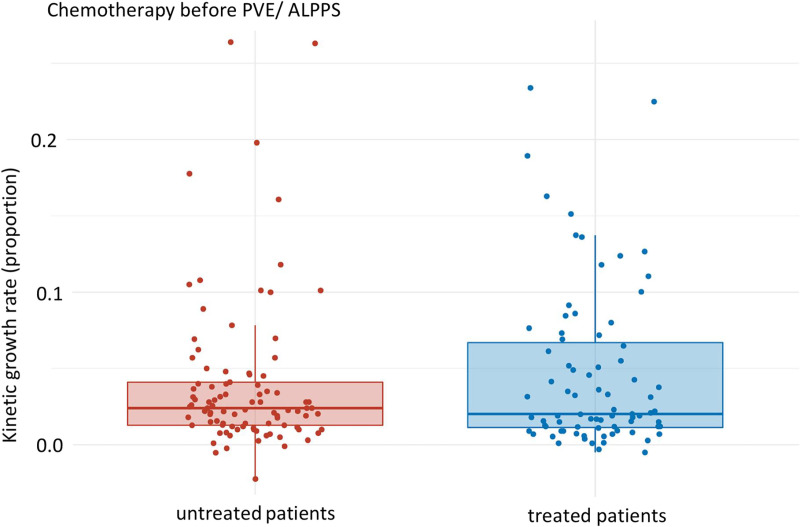
KGR of untreated patients compared to pre-intervention treated patients (PVE/ ALPPS). Median of pre-intervention untreated and treated patients: 0.024 [Q1, Q3: 0.013, 0.041] and 0.020 [Q1, Q3: 0.011, 0.067], respectively. p-value: 0.949, effect size: 0.005, magnitude: small.

Our data demonstrate that the KGR of untreated patients compared to those treated before intervention (PVE/ ALPPS) was affected: the KGR of the untreated patients (median: 0.024 [Q1, Q3: 0.013, 0.041], mean: 0.040 (SD: 0.051)) compared to the pre-intervention treated patients (median: 0.020 [Q1, Q3: 0.011, 0.067], mean: 0.046 (SD: 0.053)) decreased by about 17% on average but lacked statistical significance (p-value 0.949) (Fig 1).

**Fig 2:**
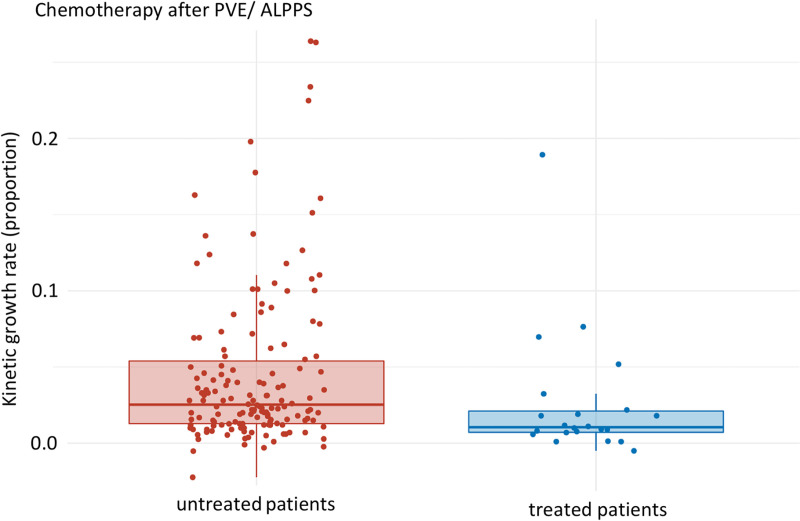
KGR of untreated patients compared to patients treated after PVE/ ALPPS in the growth interval. Median of untreated and treated patients: 0.025 [Q1, Q3: 0.013, 0.053] and 0.011 [Q1, Q3: 0.007, 0.021] respectively. p-value: 0.005, effect size: 0.217, magnitude: small.

One of the key findings of our study is presented in Fig 2. It shows the comparison of KGR between untreated patients and treated patients after intervention (PVE/ ALPPS) (median: 0.025 [Q1, Q3: 0.013, 0.053], mean: 0.045 (SD: 0.052) versus: median: 0.011 [Q1, Q3: 0.007, 0.021], mean: 0.027 (SD: 0.042)). When chemotherapy was given during growth, the negative effect on KGR was over 56%, p-value 0.005 (Fig 2).

### PVE subgroup analysis

The median KGR for patients treated with PVE was 0.02 [Q1, Q3: 0.01, 0.03].

**Fig 3:**
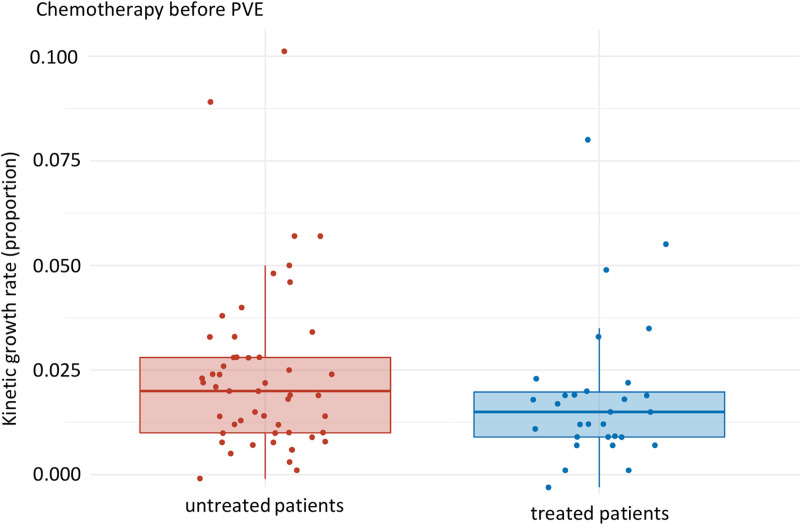
Subgroup analysis PVE only: KGR of untreated patients compared to patients treated with systemic chemotherapy prior to PVE. Median of untreated and treated patients: 0.021 [Q1, Q3: 0.011, 0.028] and 0.015 [Q1, Q3: 0.009, 0.020] respectively. p-value: 0.135, effect size: 0.165, magnitude: small.

Comparison of KGR in untreated PVE patients (median: 0.021 [Q1, Q3: 0.011, 0.028], mean: 0.024 (SD: 0.020)) to those treated with pre-intervention chemotherapy showed a negative effect of 29% (median: 0.015 [Q1, Q3: 0.009, 0.020], mean: 0.019 (SD: 0.017)), p-value 0.135 (Fig 3).

**Fig 4:**
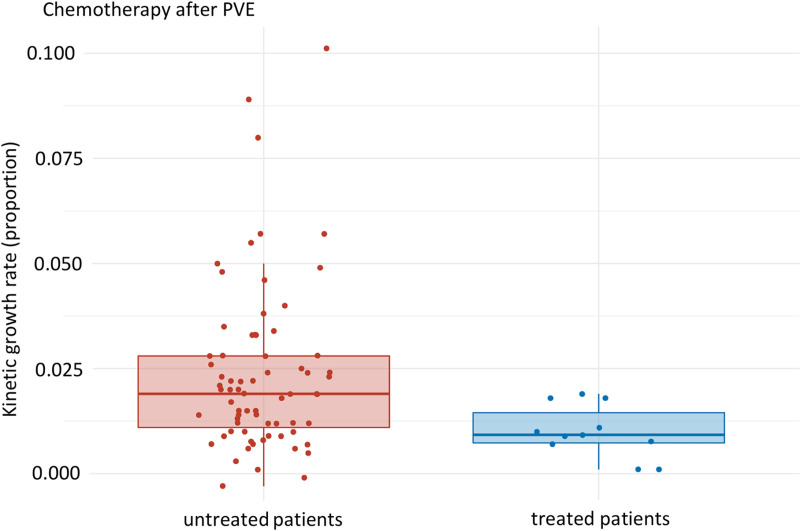
Subgroup analysis PVE only: KGR of untreated patients in comparison to treated patients after PVE. Median of untreated and treated patients: 0.019 [Q1, Q3: 0.010, 0.028] and 0.009 [Q1, Q3: 0.008, 0.015], respectively. p- value: 0.005, effect size: 0.31, magnitude: moderate.

Another key finding of our study is shown in Fig 4, which shows the effect of post-intervention chemotherapy in PVE- patients: a 47% reduction in KGR (median: 0.009 [Q1, Q3: 0.008, 0.015], mean: 0.010 (SD: 0.006)) was demonstrated compared to the untreated group (median: 0.019 [Q1, Q3: 0.010, 0.028], mean: 0.024 (SD: 0.020)), p-value 0.005 (Fig 4).

### ALPPS subgroup analysis

Analysis of patients treated with ALPPS showed a KGR of 0.04 [Q1, Q3: 0.02, 0.09], and a mean of 0.06 (Table 1).

**Fig 5:**
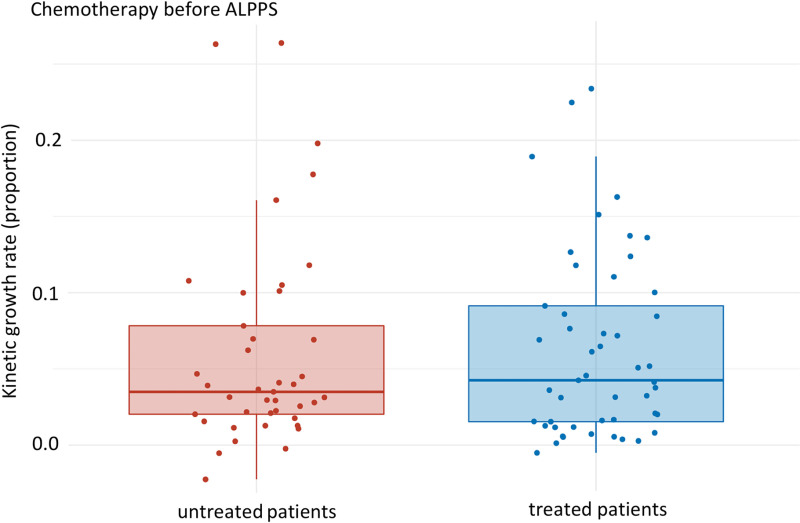
Subgroup analysis ALPPS only: KGR of untreated patients compared to treated patients before ALPPS. Median of untreated and treated patients: 0.035 [Q1, Q3: 0.019, 0.089] and 0.043 [Q1, Q3: 0.015, 0.091] respectively. p-value: 0.789, effect size: 0.029, magnitude: small.

Fig 5 shows the effect of pre-intervention chemotherapy: A 19% increase in KGR was observed in the treated patient population (median: 0.043 [Q1, Q3: 0.015, 0.091], mean: 0.063 (SD: 0.060)) compared to the untreated group (median: 0.035 [Q1, Q3: 0.019, 0.089], mean: 0.061 (SD: 0.068)), p-value 0.789 (Fig 5).

**Fig 6:**
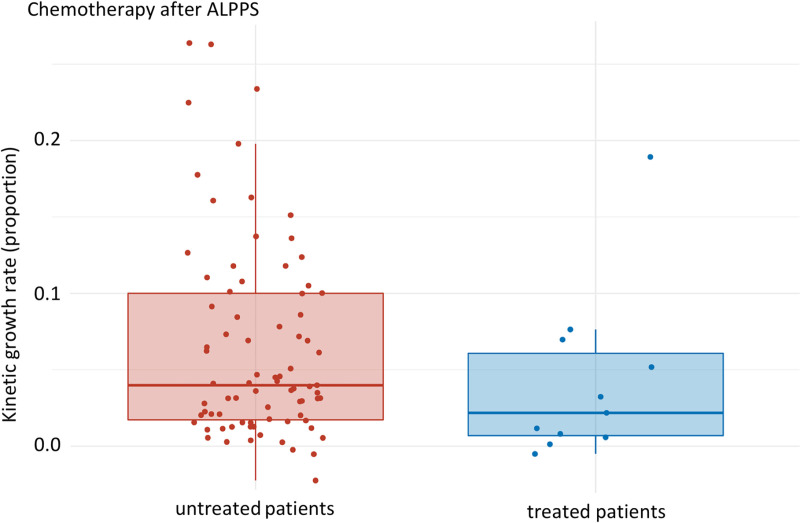
Subgroup analysis ALPPS only: KGR of untreated patients compared to treated patients after ALPPS. Median of untreated and treated patients: 0.040 [Q1, Q3: 0.017, 0.100] and 0.022 [Q1, Q3: 0.007, 0.061] respectively. p-value: 0.157, effect size: 0.15, magnitude: small.

Fig. 6 compares the KGR of the untreated group (median: 0.040 [Q1, Q3: 0.017, 0.100], mean: 0.064 (SD: 0.064)) with patients treated with post-ALPPS chemotherapy (median: 0.022 [Q1, Q3: 0.007, 0.061], mean: 0.042 (SD: 0.054)): a 45% reduction in the KGR was observed, p-value 0.157 (Fig. 6).

## Discussion

As there was no randomization with regard to ALPPS or PVE, the patient cohorts were different. For example, in the median age or gender: PVE-treated patients were five years older than the ALPPS-treated patients (effect size 0.164). There was a higher proportion of women in the PVE group than in the ALPPS group (effect size 0.082). The mean BMI of PVE (24.5) and ALPPS (24.7) were comparable (effect size 0.011).

48% of the total patient population received chemotherapy regardless of whether they received it before or after intervention, while 52% did not receive chemotherapy. Of these patients treated with chemotherapy, the majority received chemotherapy before intervention (95%). Only a small proportion were treated with chemotherapy after the intervention in the interval of hypertrophy to take place (27%). When comparing the PVE and ALPPS group, more patients in the ALPPS group received chemotherapy (PVE 39%, ALPPS 57%). A larger proportion of the PVE group did not receive any chemotherapy (PVE 61%, ALPPS 43%). In both groups of patients treated with chemotherapy, more than 90% received chemotherapy before intervention (PVE 94%, ALPPS 96%). Comparing the PVE and ALPPS group, about one third more patients in the PVE group received chemotherapy after intervention than in the ALPPS group (PVE 34%, ALPPS 22%).

The majority of the overall population received FOLFIRI (30%) or FOLFOXIRI (30%) as their chemotherapeutic agent prior to intervention. The majority of the ALPPS group received FOLFIRI (41%). Thus, the majority of PVE patients received FOLFOX or FOLFOXIRI (33% each) (effect size 0.271, medium interpretation). The most commonly used chemotherapeutic agent post-intervention was FOLFOX (50%), which was also the most commonly used agent in the ALPPS group (73%). In the PVE group, the majority received FOLFOXIRI (36%) or other chemotherapeutic agents (36%) post-intervention (effect size 0.243, medium interpretation). The interpretation of the pre- and post-interventional chemotherapy cycles was limited because the effect size was small before and tiny after intervention. One reason for this may be the small number of patients.

The histology of the primary or metastatic tumor was colorectal liver metastases in 55% of the total population, with the majority of these patients being treated with chemotherapy regardless of pre- or post-intervention (80%) and were assigned to the ALPPS group (64%). Cholangiocellular carcinoma (CCA) was diagnosed in 17% of the total population, of which 77% were assigned to the PVE group. The majority of the group diagnosed with CCA did not receive chemotherapy (92%). Other tumor entities were found in 23%.

Only a small proportion of the liver tissue examined showed parenchymal changes that could influence the FLR or KGR: fibrotic tissue (13%), steatosis tissue (9%) and cirrhotic tissue (1%), with no difference in distribution between PVE and ALPPS groups. The anatomical features of the tumor-affected liver parenchyma are another important factor that can influence the FLR/ KGR: the majority of the total population showed a multifocal distribution pattern with 62% in the PVE group and 80% in the ALPPS group. In 88% of the patient, tumor tissue was detected in the right liver lobe. Segment VIII was most affected by tumor tissue (72%), followed by segment V (66%). Comparing the PVE and ALPPS groups, the right lobe of the ALPPS group was 9% more affected by tumor tissue than the PVE group, with segment VIII remaining the most affected segment of the right lobe. In the left liver lobe, 64% of the liver was affected by tumor tissue, with segments IVa/b being the most affected (87%). When comparing the PVE and ALPPS groups, the left lobe was 15% more affected in the ALPPS group. Again, segments IVa/b were the most affected segments of the left liver lobe. A correlation between the extent of the largest tumor lesions was only possible to a limited extent (effect size 0.026, tiny).

The median of pre-intervention FLR was 19% higher in the PVE group than in the ALPPS group (effect size 0.122). Comparing the two subgroups, the KGR of the ALPPS group was twice that of the PVE group at 0.004 per week (effect size 0.414, very large). An assessment of the post-intervention chemotherapy cycles scaled to the KGR interval was limited (effect size 0.005). A comparison between the KGR of untreated patients and patients who received chemotherapy before intervention can only be assigned a low level of significance (p-value >  0.05). Even when evaluating the subgroups (PVE/ ALPPS), a comparison between untreated and treated patients before intervention cannot be considered statistically significant (p-value >  0.05). The PVE group shows a negative effect of 29%, the ALPPS group a positive effect of 19%.

The main results of our study could be visualized in the above-mentioned Figs 2 and 4: Induced hypertrophy, as measured by KGR, was negatively influenced by systemic chemotherapy when the systemic chemotherapy was given during hypertrophy, following ALPPS step one or PVE. The negative effect of chemotherapy on KGR during the growth period was seen in this study with an overall decrease of 56% in KGR (p-value 0.005); this was found to be statistically significant in the PVE group (decrease of 47%, p-value 0.005). Although a similar trend was seen in the ALPPS group, there was no statistical significance (p-value 0.157), possibly due to the small sample size.

A negative influence of chemotherapy has already been described in reviews of the general mechanism by Sharma et al. and Calistri et al. [20,21]. Also, previous concerns have been raised in the literature by Madoff and others that systemic chemotherapy at the time of growth of a future liver remnant may have a deleterious effect on the desired hypertrophy [9]. These concerns are supported by the results of this study. Our data may be in line with the work of Beal et al. who showed in a small series of 15 patients that chemotherapy after PVE did not prevent hypertrophy of the FLR in their study population, but the volume increase with chemotherapy was significantly reduced (to 89 ml, compared to a median of 135 ml without chemotherapy) (p-value 0.016) [22]. The same authors also observed that a decrease in tumor tissue size occurred [22]. Moreover, a statistically negative correlation (p-value <  0.007 by multivariate analysis) between hypertrophy and chemotherapy was also found in the larger cohort of Karsai et al., which included a total of 95 patients [23]. Moreover, the publication by Treska et al. (cohort of 38 patients with CRLM) found a negative statistical correlation between hypertrophy and chemotherapy given during the hypertrophy phase after PVE (p value < 0.003): concomitant oncological therapy increased the risk of inadequate liver hypertrophy [24]. Moreover in a cohort of 107 patients, De Beare et al. focused on the influence of platinum-based chemotherapy administered prior to PVE [25]. However this cohort did not reach statistical significance, although they found a negative effect on hypertrophy [25]. Covey et al. also speculated on the negative effect of chemotherapy prior to the hypertrophy phase induced by PVE: a reduced FLR was observed in almost all (93%) of their patients pretreated with chemotherapy prior to the hypertrophy phase [26], an effect we were not able to reproduce. In addition, animal studies have identified an influence of chemotherapy on the regeneration of liver parenchyma at the cellular level. Nithyananthan et al. reported delayed regeneration of the liver parenchyma in rats following chemotherapy administration [27]. The regeneration of liver parenchyma may be affected depending on the chemotherapy drug or combination used [28]. Gangi et al. also found chemotherapy-related liver damage, such as chemotherapy-associated steatosis or steatohepatitis, in patients with CRLM [29]. Interference with liver regeneration could negatively impact the growth of the FLR and, therefore, the KGR. This would strengthen the results of our study.

In contrast to our data, the work of Deipolyi et al. concluded that both FLR volume and KGR were only to a very limited extent affected by systemic chemotherapy. FLR growth was 6% lower in patients treated with chemotherapy, but this did not reach statistical significance (p-value 0.645) [30]. The authors concluded that “systemic therapy administered after PVE before hepatic lobectomy had no effect on FLR growth” [30]. Other observations did neither report a negative effect of chemotherapy: early work by the MD Anderson Cancer Center group, Blazer et al. examined FLR hypertrophy after PVE in patients with CRLM and showed comparable degrees of hypertrophy at four weeks (with versus without Bevacizumab: 8.8% versus 6.8%) [31]. Therefore, the data of Deipolyi et al. and Blazer et al. conflicts with our results.

However, not giving chemotherapy carries its own risks: Beal et al. showed that tumor growth occurred during the growth period when chemotherapy was not administered [22]. The fact that the number of CRLM in the liver parenchyma to be embolized influences the FLR, and thus indirectly the KGR has been described by Hayashi et al. [32]. Although no correlation could be found in our study, future confirmation of this statement would certainly be of interest. Even if the published data is heterogenous, the use of concomitant chemotherapy during the growth phase should be carefully considered, especially in patients with a small FLR and who require large hypertrophy [22,24]. Nevertheless, concomitant chemotherapy may prevent tumor progression, the formation of micro-metastases in the FLR or even remote metastases [22,24]; it may also be given to eliminate accelerated tumor growth after PVE, as has previously been reported for both primary and metastatic liver tumors [33,34].

In comparison to ALPPS, PVE treatments have a lower complication rate, as shown in a previous analysis from our centre: none of the 88 patients retrospectively analysed had a complication grade 4 or higher in Clavien-Dindo (CD) or CIRSE [12]. Published data on ALPPS procedures were associated with a potentially higher risk of complications [35]. Therefore, the individual pathway for each patient should be determined by a panel of experts from both disciplines in order to balance the higher risk of complications with the benefits of a higher KGR.

Our findings add to the existing literature as follows: Our main study results (Figs 2 and 4) support the above-mentioned literature [20,21,23,24,27], which found a negative effect of systemic chemotherapy on the liver parenchyma. Our study results also support the hypothesis of an influence of systemic chemotherapy on liver hypertrophy [9,22,25,28]. At the same time, the results of our study contradict the studies, who did not find a negative effect [30,31]. Therefore, further studies, e.g., on a larger scale or additional analysis of the types of chemotherapy, should be carried out in order to be able to make further statements about the extent and influence of chemotherapy on liver hypertrophy. Our study underlines the importance of interdisciplinary discussion, questioning and weighing up the use of systemic chemotherapy as part of the hypertrophy concept in PVE and ALLPS.

## Limitations

The results of this study are limited by the nature of this retrospective, single-center, non-randomised observation with a relatively small, and heterogeneous cohort of patients. Inclusion criteria were different for PVE and ALLPS. Furthermore, for the sake of clarity, we decided to focus on the KGR, while other parameters of hypertrophy were left aside. In some of the subgroup analyses, the number of observations was rather small. Another limitation was that patients receiving systemic therapy before and after PVE/ ALPPS were included in the subgrouping of patients treated before and after hypertrophy procedure. They were included in both subgroups by splitting the cycles of chemotherapy. The available data did not allow us to adequately analyse the effect of the tumor entity, background liver histology, tumor volume, largest liver segment/ distribution pattern, chemotherapy subtype or number of cycles of chemotherapy on the KGR.

## Clinical relevance

Chemotherapy had a negative effect on the KGR when given during hypertrophy. These data may contribute to the difficult clinical decision of whether or not to use systemic chemotherapy in this setting: to control the malignant liver lesions on the one hand, and to establish hypertrophy for safe surgery on the other.

## Abbreviations

ALPPS: Associating Liver Partition with Portal vein ligation for Staged hepatectomy
BMI: body mass index
BSA: body surface area
CCA: cholangiocellular carcinoma
CD: Clavien-Dindo
CFT: calcifying fibrous tumor
CT: computer tomography
CRLM: colorectal liver metastases
DH: degree of hypertrophy (= sFLR after PVE – sFLR before PVE)
ERH: extended right hemihepatectomy
ESL: embryonal undifferentiated sarcoma of the liver
FLR: future liver remnant
FLRV: future liver remnant volume
GIST: gastrointestinal stromal tumor
HCC: hepatocellular carcinoma
HVE: hepatic vein embolization
IQR: interquartile range
KGR: kinetic growth rate defined as the degree of hypertrophy at initial post PVE volume assessment divided by number of weeks elapsed after PVE, according to the formula KGR = degree of hypertrophy/weeks
LMS: leiomyosarcoma
ml: millilitre
N: number of members of population
PVA particles: polyvinyl alcohol particles
PVE: portal vein embolization
Q1, Q3: first quartile, third quartile
RCC: renal cell carcinoma
SD: standard deviation
SERMs: selective estrogen receptor modulator
sFLR: future liver remnant standardized to the BMI by Mosteller [16], according to the formula; sFLR% = ((FLR (cm3)/ TELV (cm3)) *100
TACE: transarterial chemoembolization
TELV: total estimated liver volume, according to the formula, TELV (cm3) = -794,41 + 1267,28 * BSA
VEGF: vascular endothelial growth factor
